# Self-Cleaning Mechano-Bactericidal Surfaces by Metal–Organic Framework Embedded Polycaprolactone Composites

**DOI:** 10.1021/acssuschemeng.5c10534

**Published:** 2026-01-30

**Authors:** Zhejian Cao, Nihal Kottan, Santosh Pandit, Jian Zhang, Maria Faresjö, Francoise M. Amombo Noa, Lars Öhrström, Ivan Mijakovic

**Affiliations:** † Department of Life Sciences, 11248 Chalmers University of Technology, Gothenburg SE-41296, Sweden; ‡ Wallenberg Initiative Materials Science for Sustainability, Department of Life Sciences, Chalmers University of Technology, Gothenburg SE-41296, Sweden; § Department of Chemistry and Chemical Engineering, Chalmers University of Technology, Gothenburg SE-41296, Sweden; ∥ Ecole des Sciences de la Santé, Université Catholique D’Afrique Centrale, B.P.1110, 11628 Yaoundé, Cameroun; ⊥ The Novo Nordisk Foundation, Center for Biosustainability, Technical University of Denmark, DK-2800 Kogens Lyngby, Denmark

**Keywords:** MOF, polycaprolactone, mechano-bactericidal surface, biofilm, biodegradable

## Abstract

Mechano-bactericidal (MB) surfaces mitigate the formation of bacterial biofilm through physical interactions without the use of antibiotics. One challenge of MB surfaces is that unremoved dead bacteria and other debris on MB surfaces could impede contact between bacteria and surface nanostructures, thus reducing their bactericidal efficiency. Herein, we report metal–organic framework (MOF)–polycaprolactone (PCL) composites as self-cleaning MB surfaces. The MIL-88B-on-UiO-66 (MoU) hybrids with nano features provide MB actions and PCL with biodegradability enables surface cleaning and refreshment. The MoU-PCL composite demonstrated effective antibacterial performance toward *Pseudomonas aeruginosa* (77.0%) and *Staphylococcus epidermidis* (89.6%) for 72 h growth. The surface degradation of MoU-PCL composites over 4 weeks confirmed the feasibility of removing surface debris and dangling MOFs to offer long-term MB performance. Our approach enables the development of MB surfaces for applications requiring a relatively long service period.

## Graphical abstract

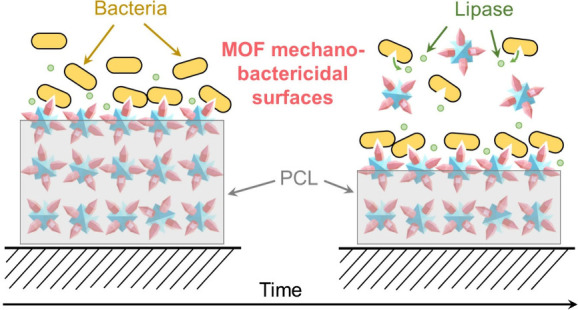

## 1 Introduction

Antibacterial surfaces are desired in various applications, such as medical devices and food packaging.^1^ Bacteria colonize a surface and form biofilms by releasing an extracellular polymeric substance (EPS) matrix comprising polysaccharides, proteins, and lipids.^2^ Bacterial biofilms have led to most nosocomial infections as they can easily grow on the surface of implants, indwelling devices, etc.^3^ Unlike the planktonic bacteria, the EPS matrix of biofilm protects the embedded bacteria against the external environment, resulting in higher resistance to conventional antibacterial strategies, such as the use of antibiotics and ionic action of metals, including silver (Ag) and copper (Cu).^4,5^ Furthermore, the misuse of antibiotics and metals could intensify antimicrobial resistance (AMR) and impact nontarget organisms in the ecosystem.^6^ According to World Health Organisation (WHO), AMR is one of the global public health threats and requires urgent countermeasures.^7^ Therefore, a sustainable and nontoxic antibacterial strategy is imperative.

Mechano-bactericidal (MB) surfaces containing nanostructures can physically rupture and destroy bacteria.^8^ MB actions involve physical interactions, and bacteria can hardly develop resistance against them.^9^ MB surfaces provide sustainable alternatives to prevent biofilm formation compared with conventional chemical approaches.^10^ Many natural MB surfaces have been studied, including cicada wings^11^ and gecko skin.^12^ Moreover, many bioinspired MB surfaces were reported based on a variety of materials, including black silicon,^13^ titania,^14^ graphene,^15^ graphite,^16^ and polycarbonate (PC).^17^ However, one major challenge of MB surfaces involves the reduction of the bactericidal efficiency when dead bacteria accumulate on the surface and cover the functional nanostructures. The debris, including dead bacteria, cannot be easily removed from certain MB surfaces without external cleaning measures. Consequently, the bactericidal performance of most MB surfaces significantly diminishes with time.^18,19^

Metal–organic frameworks (MOFs) are materials with tunable nanostructures and are often crystalline.^20,21^ MOFs are widely proposed for various applications, such as catalysts, gas adsorption, and purification, due to their high porosity and surface area, and are commercially used for gas storage and CO_2_ capture.^22–24^ Antibacterial MOFs have been reported with various antibacterial mechanisms, including (1) releasing bactericidal metal ions and organic linkers upon structure collapse, (2) releasing drugs loaded into the MOFs, and (3) photothermal and photocatalytic activities.^25–27^ However, applying MOFs as an MB surface is rarely studied since controlling the surface features, including size, pitch distance, and orientation of the MOF particles, is challenging.^28^ In our previous work, we introduced a MOF-on-MOF strategy to fabricate MOF MB surfaces using the MIL-88B­(Fe) on UiO-66­(Zr) (denoted as MoU) hybrids as the building blocks. Through an MOF-on-MOF strategy, we obtained caltrop-shaped structure offering sharp tips, spaced nanopins, and easy orientation control.^29^ These MoU surfaces demonstrated MB actions, and the scalability of these MoU hybrids allows them to be applied in real-life applications. Moreover, the synthesis temperature of MOF (<200 °C) is much lower than some of other MB surfaces, such as over 700 °C for vertical graphene through chemical vapor deposition, which could save much energy considering large-scale production.^30^ However, a drop in bactericidal efficiency was observed with bacterial growth from 24 to 72 h.^29^ Some cleaning strategies for MB surfaces require external treatment.^31^ An efficient approach for cleaning the MOF MB surfaces would provide long-term antibacterial performance, but it has not been exploited to date. We now propose that embedding layers of MoU nanostructures in a biodegradable polymer matrix could provide such a self-cleaning function, especially if the biodegradation can be triggered by the bacteria themselves.

For this purpose, polycaprolactone (PCL), a Food and Drug Administration (FDA) approved polymer with many medical applications,^32^ including sutures, wound dressing, and tissue engineering, was chosen as the matrix due to its low cost, good mechanical properties, biocompatibility, and biodegradability.^33^ PCL was reported to be degraded by bacteria, as the lipase from bacteria can hydrolyze the ester in PCL.^34–36^ Several studies on antibacterial PCL-based composites using PCL as a matrix for drugs and silver particles revealed the potential of applying PCL as the matrix for MB surfaces.^37,38^

In this work, we designed MOF-PCL composites via MOF embedding and solvent casting. The working mechanism of the designed MOF-PCL antibacterial surfaces is illustrated in Scheme 1. MOF-PCL composites with MOF nanostructures exposed on the surface act as MB surfaces. The MOF nanostructures lead to mechanical stress on bacterial membranes and lethal MB actions on the bacteria. The lipase released from the bacteria would then degrade the PCL matrix and remove debris such as dead bacteria. Subsequently, new layers of MOFs are exposed, providing fresh MB surfaces. By combining the MB actions of the MOFs and the biodegradability of the PCL, our hypothesis is that an MOF-PCL composite with self-cleaning properties will provide a relatively long-term antibacterial performance. The MOF-PCL composites were characterized by X-ray diffraction (XRD), X-ray photoelectron spectroscopy (XPS), scanning electron microscopy (SEM), and surface tension analysis. The antibacterial performance of the obtained composites was studied on Gram-positive and Gram-negative bacteria.

**Scheme 1:**
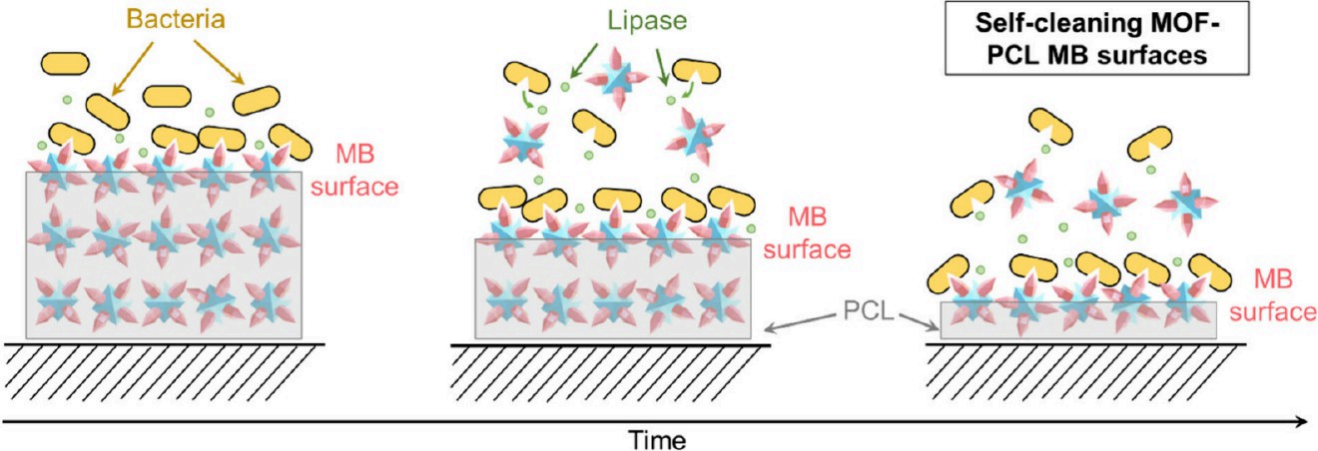
Working Mechanism of the Designed Self-Cleaning MOF-PCL Antibacterial Surfaces^a^

a MOFs such as MoU hybrids with nanostructures act as MB surfaces to rupture bacteria, and the released lipase degrades PCL, exposing fresh MOF MB surfaces.

## 2 Experimental Section

### 2.1 Chemicals and Reagents

All the starting chemicals, including polycaprolactone (*M*_w_ = 80 000) (purity ≥99.5%, Thermo Fisher), ZrCl_4_ (purity ≥99.9%, Sigma–Aldrich), Fe­(NO_3_)_3_·9H_2_O (purity ≥99.9%, Sigma–Aldrich), benzene-1,4-dicarboxylic acid (H_2_BDC) (purity ≥98%, Sigma–Aldrich), acetic acid (AcOH, purity ≥99.7%, Sigma–Aldrich), acetonitrile (CH_3_CN, purity ≥99.5%, Sigma–Aldrich), acetone (purity ≥99.5%, Sigma–Aldrich), ethyl acetate (purity ≥99.9%, Sigma–Aldrich), dichloromethane (DCM, purity ≥99.8%, Sigma–Aldrich) and *N,N*-dimethylformamide (DMF, purity ≥99.8%, Sigma–Aldrich), were purchased and used as received.

### 2.2 MOF Synthesis

UiO-66­(Zr), MIL-88B­(Fe), and MIL-88B on UiO-66 (MoU) were synthesized based on previous solvothermal synthesis methods.^29^ Briefly, UiO-66 was synthesized by loading a mixture (20 mL) of ZrCl_4_, H_2_BDC, DMF, and AcOH (with a molar ratio at 1:1:720:580) to a Teflon-lined autoclave and heated at 120 °C for 24 h. MIL-88B was synthesized by loading a mixture (5 mL) of Fe­(NO_3_)_3_·9H_2_O, H_2_BDC, DMF, and CH_3_CN (with a molar ratio at 1:1:200:290) to a microwave reaction vial (Biotage, 2–5 mL) and heating at 90 °C for 5 h. MoU was synthesized using the obtained UiO-66 as seed. 43 mg of UiO-66 in 2 mL of DMF was added to the mixture (18 mL) of Fe­(NO_3_)_3_·9H_2_O, H_2_BDC, DMF, and CH_3_CN (with a molar ratio at 1:1:200:290). After 5 min of sonication, the mixture was transferred to microwave reaction vials (Biotage, 2–5 mL) and heated at 90 °C for 5 h. After cooling the solution, the obtained powder was filtered and washed 3 times in DMF and 3 times in ethanol. The obtained MOF was dried in a static vacuum oven overnight at 60 °C before further usage. More information about the MOF synthesis can be found in section 1 of the Supporting Information (SI).

### 2.3 Fabrication of MOF-PCL Composites

#### 2.3.1 MOF-PCL Composites via MOF Embedding

##### 2.3.1.1 Fabrication of the PCL Matrix

PCL was melted in ceramic crucibles to ∼80 °C and then cast into plastic molds. The molten PCL was gently transferred to the center of the mold to avoid introducing air bubbles and prevent voids in the final product. The PCL matrices were demolded after cooling to room temperature and cut into individual PCL matrices (square 8 mm × 8 mm).

##### 2.3.1.2 MOF Embedding to the PCL Matrix

The as-obtained MoU, UiO-66, and MIL-88B were dispersed in ethanol to achieve a concentration of 5 mg/mL. After 15 min of sonication, 40 μL of MOF solution was dropcast on the PCL matrix, resulting in 200 ug/sample MOF loading. After the evaporation of ethanol at room temperature, these samples were further treated with 30 μL of acetone to embed the MOFs into the PCL. All obtained MOF-PCL samples were dried in a static vacuum oven at 40 °C overnight.

#### 2.3.2 MOF-PCL Composites via Solvent Casting

Acetone, ethyl acetate, and DCM were utilized as the solvents for the solvent casting approach. PCL (2.5 g) was dissolved in a specific solvent (5 mL) to obtain a PCL solution (500 mg/mL). For the acetone and ethyl acetate samples, a warm water bath (40 °C) was used to accelerate the dissolution of the granules. The as-obtained MoU, UiO-66, and MIL-88B were dispersed in the corresponding solvent (10 mg/mL) with 10 min of sonication. Thereafter, different volumes (0, 0.5, 1 mL) of the MOF suspension were mixed with the PCL solution (1 mL) with 10 min of sonication to achieve MOF-PCL solutions with different MOF-to-PCL ratios (0, 1, 2% (w/w)). Thereafter, 50 μL of MOF-PCL solution was drop-cast to a stainless-steel round slice (diameter 10 mm). After evaporation of the solvent at room temperature, we obtained MOF-PCL composites through a solvent casting approach. All obtained MOF-PCL samples were dried in a static vacuum oven at 40 °C overnight.

### 2.4 Material Characterization

#### 2.4.1 Scanning Electron Microscopy (SEM)

The surface structures of all samples were characterized using SEM (JEOL, Model JSM-7800F Prime) with an accelerating voltage of 5 kV. All bacteria-attached samples were treated with 3% glutaraldehyde for 2 h and then dehydrated by an ethanol dilution series (40%, 50%, 60%, 70%, 80%, and 90%) for 10 min each and then with absolute ethanol for 15 min. All samples were dried under a vacuum for 3 h at 40 °C and then coated with 15 nm gold to provide good conductivity.

#### 2.4.2 X-ray Diffraction (XRD)

The XRD spectra of the PCL and MOF-PCL composite substrates were characterized using a Bruker XRD D8 Advance with Cu Kα X-ray source (λ = 1.54 Å) at room temperature with a 2θ scanning range of 5°–40°. Simulated XRD patterns of the MOFs were calculated using Mercury software with crystal data from the Cambridge Structure Database (CSD).^39^

#### 2.4.3 X-ray Photoelectron Spectroscopy (XPS)

The surface chemistry of the obtained MOF samples was investigated with a PHI VersaProbe III X-ray photoelectron spectroscopy (XPS) instrument with an Al Kα X-ray source. The data was analyzed with CasaXPS software.

#### 2.4.4 Contact Angle (CA) Measurements

The surface wettability of PCL and MOF-PCL composite substrates was characterized by measuring the water contact angle under atmospheric conditions using an optical tensiometer (Attension, Biolin Scientific). The images for analysis were taken within 5 s of the droplets (Milli-Q water) being dispensed on the surface. The result was taken from an average of 3 replicates.

#### 2.4.5 Atomic Force Microscopy (AFM)

The surface topography of PCL after degradation was characterized using AFM (NTEGRA Probe Nanolaboratory). Tests were recorded in tapping mode with images at a scan rate of 0.5 Hz. Three representative places were measured for each sample and used for the root-mean-square (RMS) roughness calculation using the Gwyddion software.

### 2.5 Assessment of Antibacterial Performance and Biodegradation

#### 2.5.1 Bacterial Viability

Plate counting of the colony-forming unit (CFU) experiment was utilized to evaluate the bacterial viability as reported in our previous studies.^40,41^ *Pseudomonas aeruginosa* (*P. aeruginosa*, PA01) and *Staphylococcus epidermidis* (*S. epidermidis*, ATCC 35984) were obtained from the Gothenburg University Culture Collection (CCUG), and these were used to study the viability of Gram-negative and Gram-positive bacteria, respectively. A single colony of each species was incubated in a liquid medium, Lysogeny broth (LB) for *P. aeruginosa* and tryptic soy broth (TSB) for *S. epidermidis*, at 37 °C overnight (16 h). A 25 μL portion of the obtained culture was added to 5 mL of fresh medium to obtain an inoculum containing (2–5) × 10^6^ CFU/mL bacteria. 60 μL of the obtained inoculum was dispensed onto the test surfaces (square, 8 mm × 8 mm). The empty plate wells and gaps were filled with sterilized water to prevent the evaporation of the inoculum media. After bacterial growth at 37 °C for 24 and 72 h, the media were gently extracted to not disturb the biofilm. The attached biofilm was collected from the surface and homogenized using sonication (Digital Sonifier, Branson, 10% amplitude, 30 s) in 5 mL of 0.89% NaCl. The homogenized suspensions were diluted serially (×10) and plated into agar plates. The plates were incubated at 37 °C for 24 h and the number of colonies on the agar plates was counted. The number of viable bacteria (CFU/sample) was estimated by the number of colonies counted in plates and their corresponding dilution factors. The bactericidal efficiency was obtained by normalizing the CFU counts of each of the MOF-PCL composite surfaces to that of the PCL surface. The experiments were conducted with three biological replicates, and the mean values ± standard deviation are reported. The statistical significance between the MoU-PCL surface and PCL surface was examined using an independent *t*-test and differences between groups were considered statistically significant at (*) *p* < 0.05, (**) *p* < 0.01, and (***) *p* < 0.001.

#### 2.5.2 Fluorescence Microscopy

The live/dead assay was performed by using fluorescence microscopy. The bacteria-attached (24 h growth) samples were stained with LIVE/DEAD BacLight bacteria viability stains kit L7012 (Invitrogen, Molecular Probes, Inc. Eugene, OR, USA). Green-fluorescent nucleic acid stain SYTO 9 and red-fluorescent nucleic acid stain propidium iodide (PI) were utilized to mark the live and dead bacteria, respectively. The biofilms were stained for 20 min with SYTO 9 and PI, and fluorescence microscopy images were obtained using a Zeiss microscope (Leica CTR4000).

#### 2.5.3 Biodegradation of PCL and MoU-PCL Composites

To study the biodegradation properties, samples (square, 8 mm × 8 mm) of PCL and MoU-PCL composites were loaded into a culture tube with 5 mL of bacterial inoculum containing (2–5) × 10^6^ CFU/mL *P. aeruginosa*. The control samples were loaded into a culture tube with 5 mL LB media. All samples were incubated at 37 °C. After incubated for 3 days, the culture media was changed every 48 h to provide enough nutrients. The samples were characterized by SEM after 2 and 4 weeks. To verify the mechanism of degradation, the samples were also tested with lipase (lipase obtained from Porcine Pancreas, Tokyo Chemical Industry Co., Ltd.) and *Escherichia coli* (*E. coli*, UTI89) with the same protocol (section 2 in the SI).

## 3 Results and Discussion

We applied two fabrication strategies to achieve MOF-PCL composites: MOF embedding and solvent casting. In the MOF embedding approach (Figure 1a), the PCL beads were melted and transferred to the mold to form PCL matrices by cooling at room temperature. Due to excellent fluidity, molten PCL could be easily shaped into designed structures (section 3 in the SI). After that, MOFs were drop-cast onto the PCL matrices, followed by acetone surface treatment to embed the MOFs into the PCL, as other test solvents could lead to irregular surfaces (section 4 in the SI). Through the MOF embedding method, we obtained MOF-PCL composites with exposed MOF nanostructures on the surface. No significant decrease in mechanical performance was observed after the MOF embedding treatment (section 5 in the SI).

**Figure 1:**
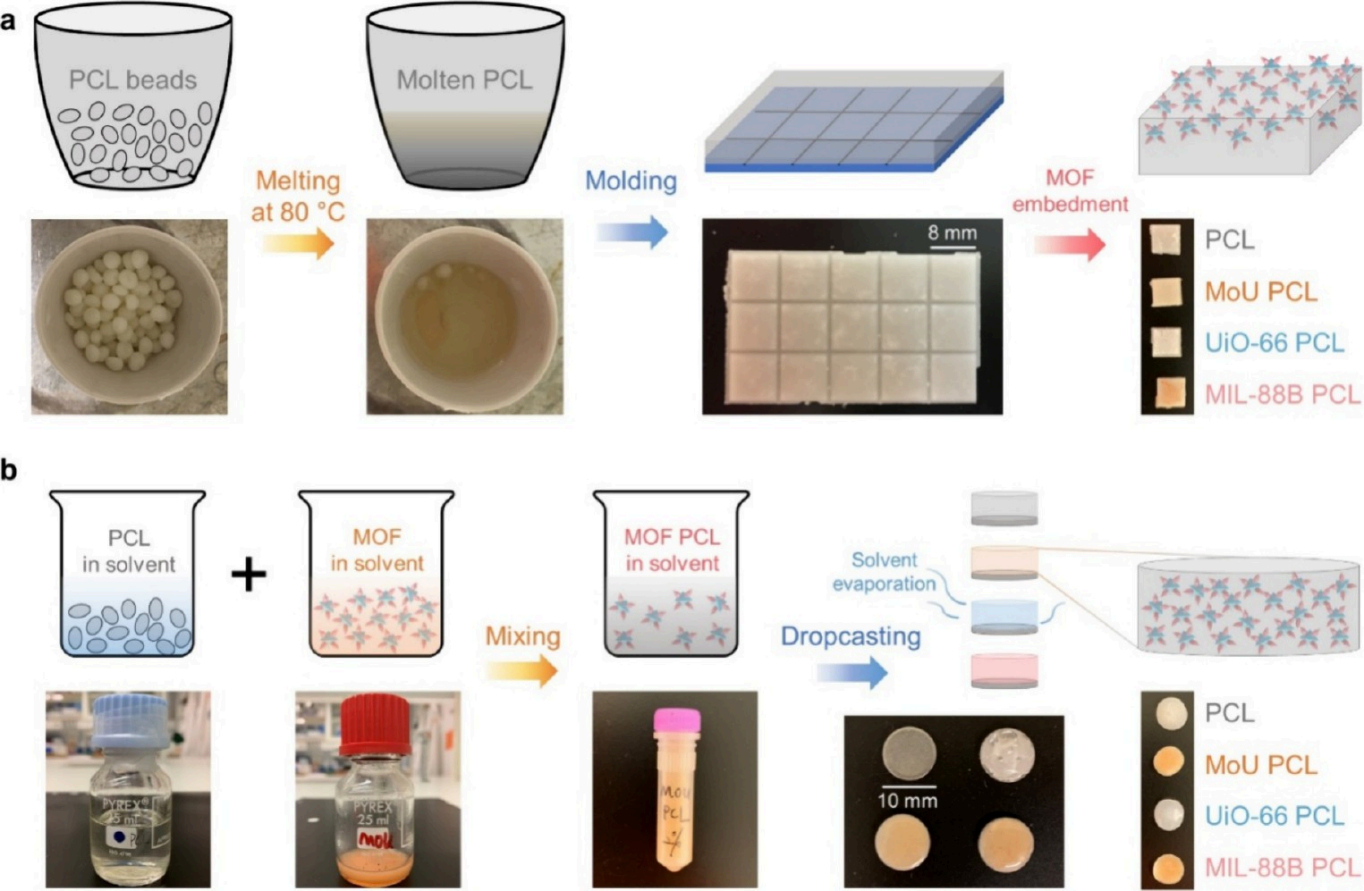
(a) MOF embedding approach to fabricating MOF-PCL composites. Molten PCL was cooled and solidified in a mold to obtain PCL matrices, following the embedment of MOFs. (b) Solvent casting approach to fabricate MOF-PCL composites. PCL and MOFs were mixed in specific solvents and then dropcast onto stainless steel slices. After the evaporation of solvent, MOF-PCL composites were obtained.

In contrast, the solvent casting method could provide relatively homogeneous MOF-PCL composites. As illustrated in Figure 1b, PCL and MOFs were mixed in specific solvent, such as acetone and ethyl acetate, by stirring and then dropcast onto stainless steel slices.^42^ After evaporation of the solvent, we obtained MOF-PCL composite films. Notably, most MOFs were immersed in the PCL matrix, and the nanostructures were hardly exposed on the surface through the solvent casting method. Without the exposed nanostructures, no direct MB action would be expected. Furthermore, some voids and cracks were observed on the surface during solvent evaporation, which could impact on the quantitative study of bactericidal efficiency (section 6 in the SI). Therefore, the antibacterial performance was investigated only with the MOF-PCL composites fabricated by the MOF embedding method in this work.

The microstructure of the obtained samples was characterized by using SEM. As shown in Figure 2a, the intact PCL matrix presented a smooth morphology. MoU particles presented a caltrop-like shape, consisting of octahedral UiO-66­(Zr) and bipyramidal hexagonal prism MIL-88B­(Fe). The obtained MOFs possessed sharp features in the nanoscale range, e.g., the diameter of the MIL-88B tips was less than 5 nm, suitable for MB actions. Notably, MoU showed sharp tips in random directions when dropcasting, while the control sample with MIL-88B had the needles laid down horizontally, with most sharp tips inaccessible to bacteria.

**Figure 2:**
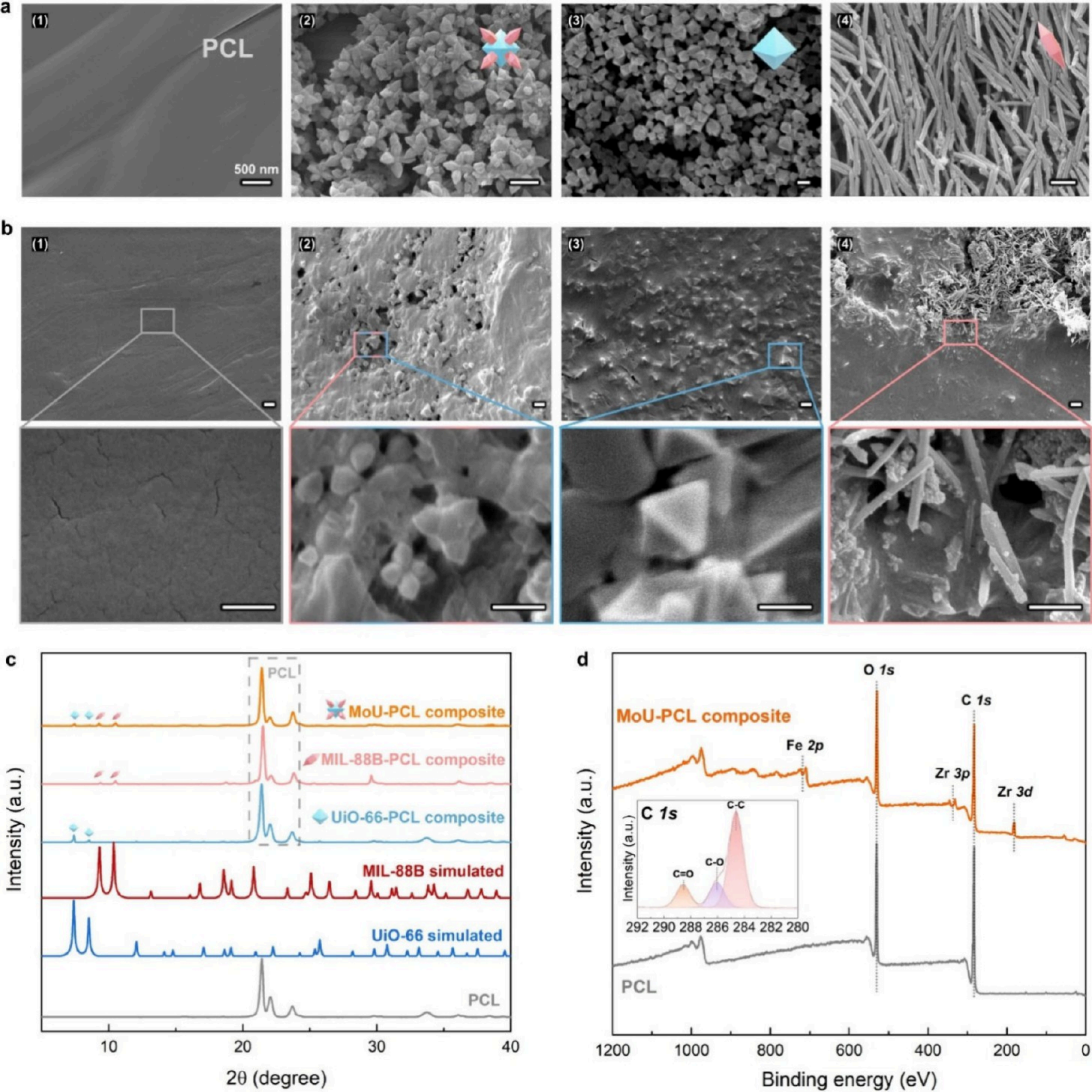
(a) SEM images of (1) intact pure PCL, (2) MoU, (3) UiO-66, and (4) MIL-88B; (b) SEM images of acetone treated (1) pure PCL, (2) MoU-PCL composite, (3) UiO-66-PCL composite, and (4) MIL-88B-PCL composite. Scale bar for SEM images = 500 nm. (c) XRD patterns of simulated UiO-66, MIL-88B, and experimental results of pure PCL and MOF-PCL composites, with characteristic peaks of UiO-66 and MIL-88B marked and PCL peaks in the gray dashed frame. (d) XPS spectra of PCL and MoU-PCL composites, with zoomed-in, high-resolution XPS spectra for C 1s.

After acetone treatment, as demonstrated in Figure 2b, the pure PCL matrix maintained its smooth morphology, while in the MOF-PCL composites the MoU embedded in the matrix presented exposed sharp nanostructures on the surface. Clearly, Figure 2b shows that the MOFs maintain their characteristic morphology, indicating that the MOF embedding process and the PCL matrix did not damage the MoU structures. The XRD patterns in Figure 2c confirm the presence of the corresponding MOF crystals in the composites. All MOF composites showed prominent PCL peaks (around 21°) and relatively weak MOF peaks (7.4°, 8.5° for UiO-66 and 9.3°, 10.4° for MIL-88B), as PCL matrices were much thicker than the MOF-embedded regime. The XPS spectra confirmed the composition of PCL (O 1s and C 1s)^43^ (section 7 in the SI), Zr^4+^ (Zr 3p and Zr 3d, from UiO-66), and Fe^3+^ (Fe 2p, from MIL-88B), as seen in Figure 2d. The maintenance of the geometric features and crystallinity of the MOFs in the composites was essential for effective MB actions.

The surface tension of the obtained MOF-PCL composites was measured by a water contact angle (CA) experiment. MoU-PCL composites (72° ± 5°) demonstrated a close water CA value similar to the pristine PCL (74° ± 2°), which we hypothesize is due to the PCL shielding the MoU after the MOF embedding (section 8 in the SI).

To investigate the bactericidal efficiency of MOF-PCL composites, we applied plate counting of colony-forming units (CFUs) and live/dead staining methods, using *Pseudomonas aeruginosa* (*P. aeruginosa*) and *Staphylococcus epidermidis* (*S. epidermidis*) as representative organisms for Gram-negative and Gram-positive bacteria, respectively. According to the CFU results (Figure 3, section 9 in the SI), MoU-PCL composites presented prominent antibacterial performance (loss of viability for 24 h at 98.8% for *P. aeruginosa* and 93.6% for *S. epidermidis*). MoU-PCL composites demonstrated higher bactericidal efficiency than UiO-66-PCL and MIL-88B-PCL composites, which could be attributed to the eight sharp tips in MoU, where there is no sharp tip structure in UiO-66 and only two sharp tips in MIL-88B. Furthermore, due to the multidirectional orientation of the tips in MoU, MoU-PCL composites possessed more accessible tips interacting with bacteria. Notably, the two tips of MIL-88B are in line, pointing in two opposite directions, so only one tip would be exposed as an MB structure in the embedded composites.

**Figure 3:**
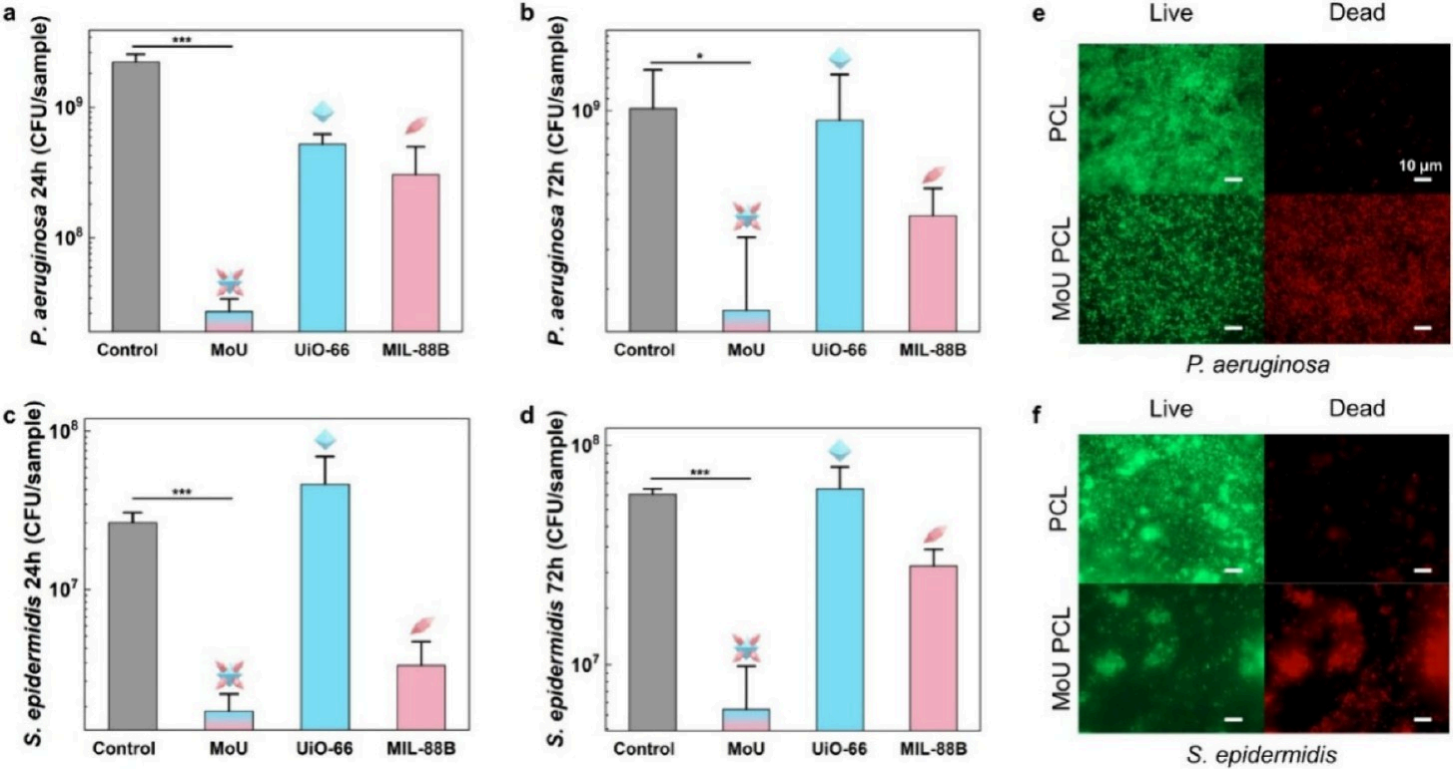
CFU results of biofilms on MOF-PCL composites (a) 24 h growth of *P. aeruginosa*, (b) 72 h growth of *P. aeruginosa*, (c) 24 h growth of *S. epidermidis*, (d) 72 h growth of *S. epidermidis*, data represent the mean ± standard deviation of three biological replicates ((*) *p* < 0.05, (***) *p* < 0.001). Live/dead fluorescent staining images of attached bacteria of 24 h growth on PCL and MoU-PCL composites with (e) *P. aeruginosa* and (f) *S. epidermidis*, green indicating live bacteria and red indicating dead bacteria, scale bar = 10 μm.

Our previous work has demonstrated that the Gram-positive bacteria could be impacted both mechanically and chemically by MIL-88B, as the Gram-positive bacteria are more sensitive to Fenton-like reactions resulting from the released Fe ions from the MIL-88B. Consequently, a relatively high loss of viability (87.5%) for 24-h-old *S. epidermidis* was found in the MIL-88B-PCL composite. The bactericidal efficiency of MoU-PCL composites was also observed in the live/dead fluorescent staining images (Figure 3e and 3f). Relatively dense live attached bacteria (green signals) and no significant dead bacteria (red signals) were seen in PCL samples for both *P. aeruginosa* and *S. epidermidis*. In contrast, less densely attached bacteria (green signals) and many dead bacteria (red signals) were found in the MoU-PCL composites. This aligns with the CFU results.

The interaction between the MOF-PCL composites and bacteria was characterized by SEM, as shown in Figure 4. Degradation of the PCL was observed, where the smooth PCL surfaces became pleated, as highlighted by the yellow-dashed ellipses. This pleated morphology was also observed in the PCL samples immersed in a lipase solution for 24 h. However, when grown with low intrinsic lipase-active strain *E. coli* for 24 h, no pleated structure was found (SI, section 2). These results support our hypothesis on the biodegradation of the PCL by the lipase release by the bacteria.^34,44^ *P. aeruginosa* and *S. epidermidis* formed dense biofilms within 24 h on PCL surfaces with their bacterial envelopes intact (Figure 4a and 4c). However, on the MoU-PCL composite, the attached bacteria were in disintegrated clusters, and the bacterial envelopes were deformed due to the nanotips of MoU, as highlighted in the red dashed rectangles. Particularly, the nanotips of the MOFs could directly penetrate the bacterial cell membrane and destroy the bacteria by impaling, or they can cause mechanical injury by deforming the bacterial envelope (Figure 4b and 4d). These deformations by nanostructures were reported to be capable to lead to apoptosis-like death.^45,46^ These findings support the notion that MoU-PCL composites presented bactericidal efficiency higher than that of UiO-66-PCL and MIL-88B-PCL composites, as MoU had more tips with multiple pointing directions. These MB mechanisms have been found when directly using MOFs in our previous work,^29^ and they were successfully observed in these MOF-PCL composites, confirming that the MOF-PCL composites maintained MB actions over time to prevent the formation of biofilm. The embedment of the MOF into PCL did not eliminate the critical features of the MOF, allowing MOF-PCL composites for MB applications.

**Figure 4:**
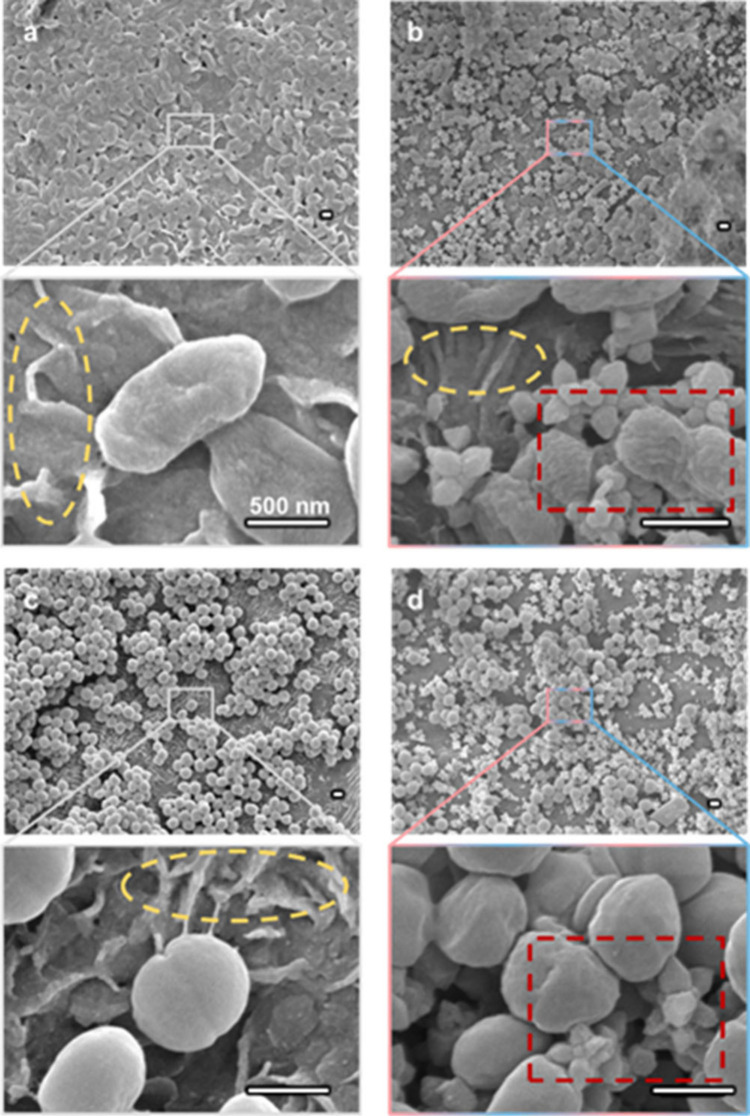
SEM images of attached bacteria after 24 h growth on different surfaces. (a) *P. aeruginosa* on PCL, (b) *P. aeruginosa* on MoU-PCL composites, (c) *S. epidermidis* on PCL, (d) *S. epidermidis* on MoU-PCL composites. The deformation of the bacteria resulting from the MOFs is highlighted in the red-dashed rectangles. The degradation of PCL is highlighted in the yellow-dashed ellipses. Scale bar = 500 nm.

To investigate relatively long-term antibacterial performance, bacteria were grown for 72 h by exchanging fresh media every 24 h to avoid a lack of nutrients. According to the CFU results (Figure 3b and 3d), MoU-PCL composites presented the highest bactericidal efficiency (77.0% for *P. aeruginosa* and 89.6% for *S. epidermidis*) among the tested samples. Yet, it was slightly reduced compared to dealing with 24 h growth. One potential explanation would be that the degradation of PCL within a static environment would lead to an unsmooth surface (section 2 in the SI), as we observed that the RMS roughness of the lipase-degraded PCL is higher than that of the nondegraded PCL, according to the AFM results. This suggests heterogeneous degradation on the PCL surface. We hypothesize that the nonuniformed degradation of PCL and random distribution of MOFs could lead to incomplete exposure of MOF structures in certain regions, resulting in a diminished MB performance. This might also explain why the standard deviations of the CFU results have increased from 24 h to 72 h growth, as the degradation of PCL led to nonuniform surfaces.

One advantage of MOF-PCL composites superior to the sole MOF particles is their self-cleaning property, where biodegradability is the key mechanism enabling long-term antibacterial performance. To verify the biodegradability of the PCL and MOF-PCL composites, the microstructures were analyzed after 2- and 4-week immersion in LB media (culture media changed every 48 h) with and without *P. aeruginosa*, as demonstrated in Figure 5. The PCL maintained its smooth surface after 2- and 4-week immersion in media without bacteria (Figure 5a and 5b), confirming that the PCL could sustain its structure and protect the immersed substances when it is not employed in a microbial environment. However, when *P. aeruginosa* was introduced, the PCL surfaces after sonication to remove the attached bacteria showed pleated morphology (Figure 5c and 5d), which was attributed to the biodegradation by the depolymerase such as lipase from the bacteria.^35,47^ Similar biodegradation of PCL was found in MoU-PCL composites, as shown in Figure 5ef. Apart from the pleated surface caused by biodegradation, plenty of pits of less than 500 nm were observed on the MoU-PCL composites. The shape of the pits corresponded to the MoU, indicating that the dangling MoU particles were removed with the degradation of PCL. Notably, when same treatment applied to MoU-PCL samples grown with low intrinsic lipase-active strain *E. coli*, no characteristic pits were observed after 2 weeks (SI, section 2). These results revealed the possibility of refreshing the surfaces via the biodegradation of the PCL matrix. The degradation of MOF particles was observed, as MoU were also reported to be degradable after 7 days in culture media, which would be good for drug delivery applications.^48^ The degradation results confirmed the self-cleaning performance of the MoU-PCL composite.

**Figure 5:**
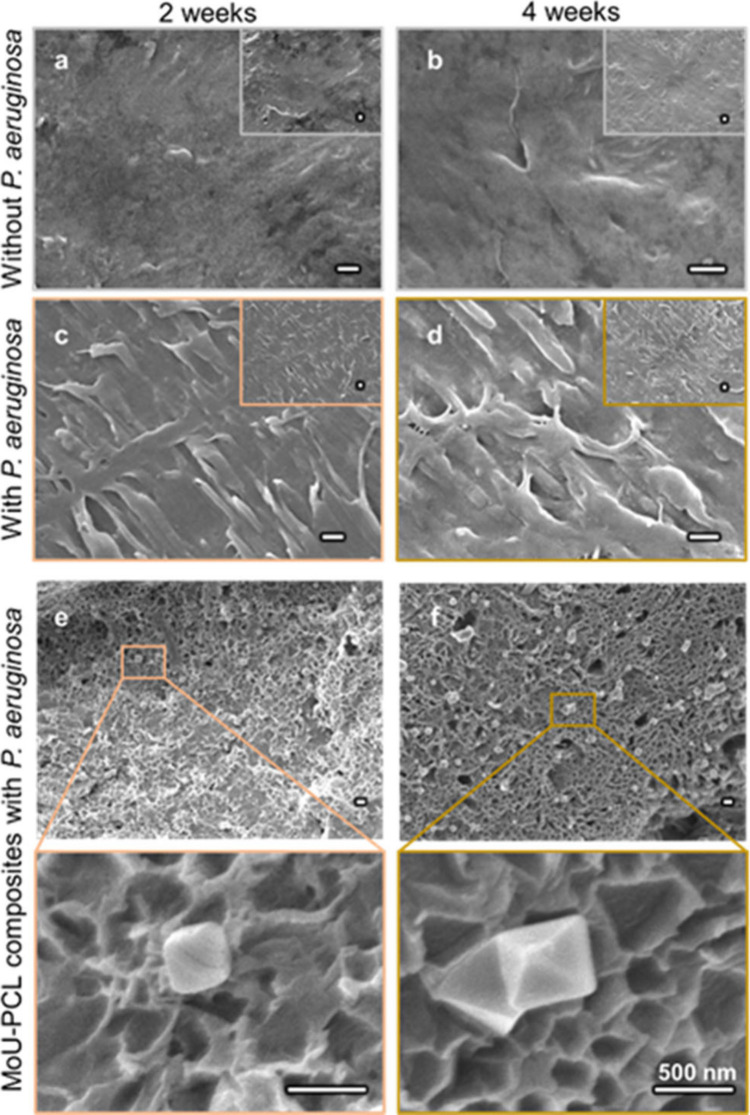
(a-d) SEM images of PCL in LB media at 37 °C for 2 and 4 weeks with and without *P. aeruginosa*. Degradation of PCL was found in the presence of *P. aeruginosa*. SEM images of MoU-PCL composites in LB media at 37 °C with and without *P. aeruginosa* for 2 weeks (e) and 4 weeks (f). Scale bar = 500 nm.

Even though PCL, MIL-88B­(Fe), and UiO-66­(Zr) have been reported with no/low cytotoxicity,^32,49^ more assessment might require for certain applications. For example, marine relevant organisms, marine mimic culture could be applied for the antifouling applications.^50,51^ We tested the cytocompatibility of the MoU structure (SI, Section 10). However, in-depth *in vivo* studies are required before use in medical applications, especially considering the optimization of the mechanical durability, further biocompatibility testing, degradation kinetics of PCL, ratio of MOF loading, degradation of the MOFs and potential release of Fe and Zr ions.^52,53^

## 4 Conclusions

In conclusion, we demonstrated that MOF-PCL composites could act as self-cleaning surfaces to provide long-term antibacterial protection. Two approaches to fabricating MOF-PCL composites were applied, MOF embedding and solvent casting. The MOFs maintained their crystallinity and morphology after the embedment. The MoU-PCL composites presented prominent antibacterial performance, due to having more accessible nanostructures to bacteria. Direct impaling and mechanical injury by the nanostructures were observed in both *P. aeruginosa* and *S. epidermidis*, confirming that the MoU-PCL composites could be applied as mechano-bactericidal (MB) surfaces. Biodegradation of PCL and MoU-PCL was observed in the presence of *P. aeruginosa*. The degradation enabled the removal of debris and dangling MOFs to offer long-term efficacious MB surfaces. This work offers new sights in dealing with one challenge of MOF MB surfaces in refreshing the nanostructures to maintain bactericidal efficiency. We believe our findings could inspire more self-cleaning MB surface designs for specific applications such as marine antifouling coatings and medical devices.

## Supplementary Material



## Notes

Details of MOF synthetic procedures, morphology of solvent casting samples, biodegradation of PCL by lipase, wettability, and supporting references (PDF)

## ABBREVIATIONS

MOF: metal–organic framework
PCL: polycaprolactone
XRD: X-ray diffraction
XPS: X-ray photoelectron spectroscopy
SEM: scanning electron microscopy
AFM: atomic force microscopy
RMS: root-mean-square
MoU: MIL-88B-on-UiO-66
MB: mechano-bactericidal
EPS: extracellular polymeric substance
AMR: antimicrobial resistance
FDA: Food and Drug Administration
LB: Luria–Bertani
CFU: colony forming unit
