# Supplementary material for: Self-Cleaning Mechano-Bactericidal Surfaces by Metal–Organic Framework Embedded Polycaprolactone Composites
Source: ACS Sustain Chem Eng. 2026 Jan 30;14(10):4787–97. doi: 10.1021/acssuschemeng.5c10534 (PMC12997249; doi:10.1021/acssuschemeng.5c10534)
Supplement: Supplementary file 1 [file sc5c10534_si_001.pdf]

Supporting Information

Self-Cleaning Mechano-Bactericidal Surfaces by Metal-Organic Framework Embedded Polycaprolactone Composites

Zhejian Cao[1,2]*, Nihal Kottan[1], Santosh Pandit[1], Jian Zhang[1], Maria Faresjö[1], Francoise M. Amombo Noa[3,4], Lars Öhrström[3], and Ivan Mijakovic[1,2,5]

[1]Department of Life Sciences, Chalmers University of Technology, Gothenburg SE-41296, Sweden.
[2]Wallenberg Initiative Materials Science for Sustainability, Department of Life Sciences, Chalmers University of Technology, Gothenburg SE-41296, Sweden.
[3]Department of Chemistry and Chemical Engineering, Chalmers University of Technology, Gothenburg SE-41296, Sweden
[4]Ecole des Sciences de la Santé, Université Catholique D'Afrique Centrale, B.P.1110 Yaoundé, Cameroun
[5]The Novo Nordisk Foundation, Center for Biosustainability, Technical University of Denmark, DK-2800 Kogens Lyngby, Denmark

E-mail: zhejian@chalmers.se

Contents

Section 1: Metal-organic framework (MOF) synthesis 2
Section 2: Biodegradation of PCL investigation 3
Section 3: Fabrication of PCL into different structures 4
Section 4: MOF embedding with different solvents 5
Section 5: Mechanical test of the PCL and MoU-PCL specimen 6
Section 6: Microstructure of MOF-PCL composites via solvent casting 7
Section 7: High-resolution XPS spectra for O *1s* and C *1s* 8
Section 8: Water contact angle measurement 9
Section 9: Colony forming unit (CFU) results 10
Section 10: MoU cytotoxicity on human cancer cells 11
Supporting references 12

Section 1: Metal-organic framework (MOF) synthesis

MOF synthesis:

UiO-66(Zr), MIL-88B(Fe), and MIL-88B on UiO-66 (MoU) were synthesized based on previous methods,[1] as listed below.

UiO-66 was synthesized using a previously reported method[2]. H_2BDC solution (in DMF, 36 mM) and $ZrCl_4$ solution (in DMF, 36 mM) were mixed. AcOH was then added to this solution and stirred for 10 min to thoroughly mix the solutions, at room temperature. The molar ratio of $ZrCl_4$: H_2BDC : DMF: AcOH was 1:1:720:580. The mixture was poured into a Teflon-lined autoclave and heated at 120 °C for 24 h. After cooling the solution, the obtained powder was filtered and washed 3 times in DMF and 3 times in ethanol. The obtained powder was then dried in a static vacuum oven overnight at 60 °C.

MIL-88B was synthesized using a previously reported method[3]. A mixture of H_2BDC solution (in DMF, 100 mM) and $Fe(NO_3)_3{\cdot}9H_2O$ solution (in DMF, 200 mM) was prepared, and CH_3CN was added to this solution and stirred for 10 min at room temperature. The molar ratio of $Fe(NO_3)_3{\cdot}9H_2O$: H_2BDC : DMF: CH_3CN was 1:1:200:290. 5 mL of the resultant mixture was heated in a microwave reaction vial (Biotage®, 2-5 mL) and heated to 90 °C for 5h. After cooling to room temperature, the obtained particles were filtered and washed 3 times in DMF and then 3 times in ethanol. The obtained powder was dried in a static vacuum oven overnight at 60 °C.

MoU was synthesized using previously reported methods[3,4]. A mixture of H_2BDC solution (in DMF, 100 mM) and $Fe(NO_3)_3{\cdot}9H_2O$ solution (in DMF, 200 mM) was prepared, and CH_3CN was added to this solution and stirred for 10 min at room temperature. 43 mg UiO-66 (in 2 mL DMF), prepared using the method stated above, was added to this mixture and was sonicated for 5 min. 5 mL of the resultant mixture was transferred to a microwave reaction vial (Biotage®, 2-5 mL) and heated to 90 °C for 5h. After cooling to room temperature, the resultant powder was filtered and washed 3 times in DMF and then 3 times in ethanol. The obtained powder were dried in a static vacuum oven overnight at 60 °C.

Section 2: Biodegradation of PCL investigation

To understand the mechanism of degradation of PCL and MoU-PCL composite, the samples (square, 8 mm *8 mm) were loaded to 5 mL phosphate buffered saline (PBS, pH 7, Gibco) containing 5 mg lipase, and 5 mL bacterial inoculum containing 2 - 5 × 10^6 CFU/mL *E. coli*., respectively. Control samples were loaded to 5 mL PBS and LB media, respectively. All samples were incubated at 37 °C. lipase solution and LB media were changed every 48 h to provide enough active lipase and nutrients. In **Figure S1ab**, we notice that after 24 h pleated morphology was observed on the PCL sample in lipase solution, while smooth morphology was maintained in the samples in PBS solution. This is consistent with the observation in main text (Figure 4 and 5), suggesting the degradation of PCL is from the lipase.[5] The atomic force microscopy (AFM) mapping (**Figure S1cd**) revealed the increase of the root mean square (RMS) roughness of the PCL with lipase (30 ± 6 nm) compared with in intact PBS solution (15 ± 2 nm). *E. coli* (UTI89) has low intrinsic lipase activity. No pleated structure was observed on the PCL surface after 24h growth with *E. coli* (**Figure S1e**), suggesting no/low degradation of PCL. Moreover, no characteristic triangle pits were found in the sonicated MoU-PCL samples with 2-week growth of *E. coli* (**Figure S1f**), showing no significant self-cleaning phenomenon compared to *P. aeruginosa* in main text Figure 5.

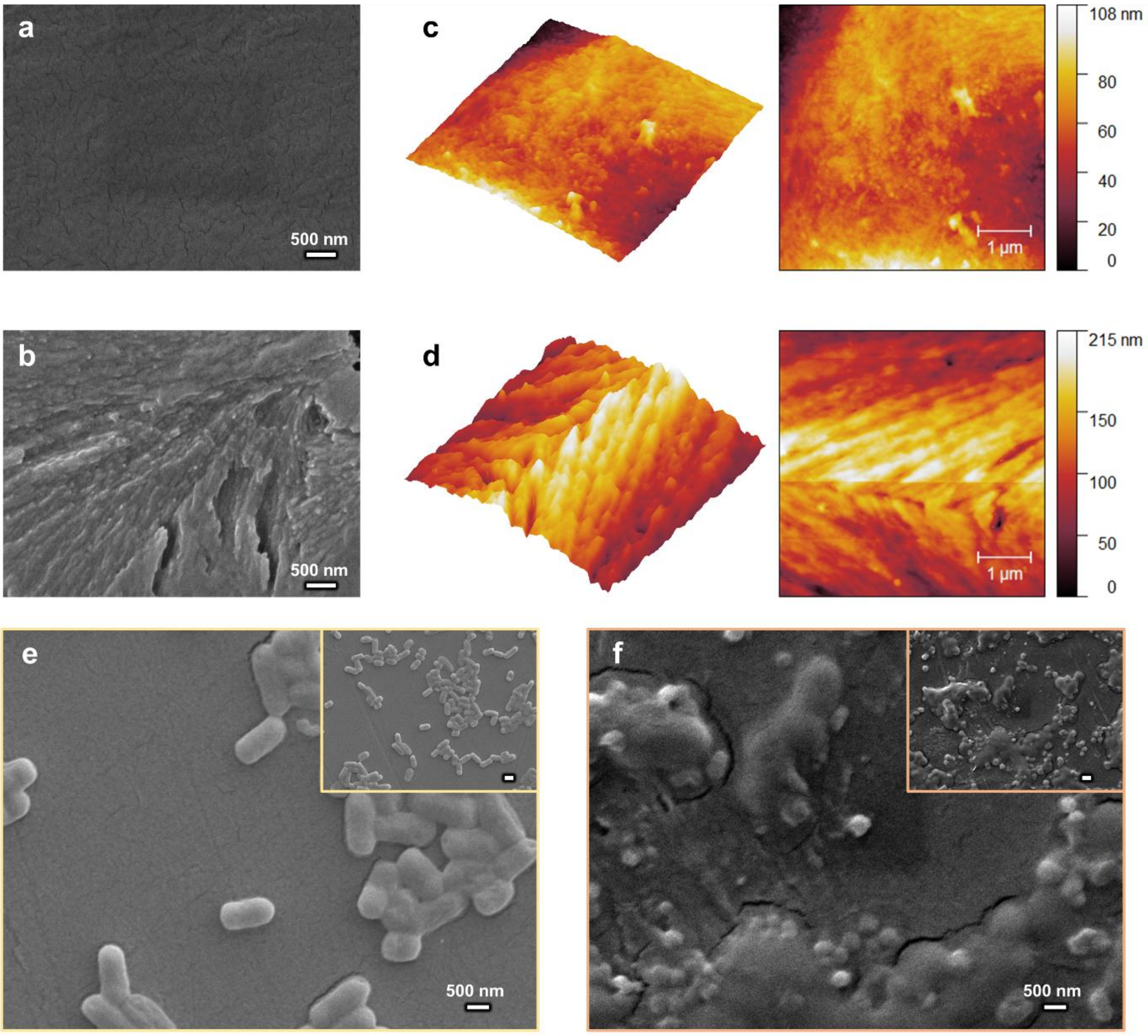

Figure S1 SEM images of PCL after 24 h in (a) PBS, and (b) lipase solution. AFM mapping of PCL after 24 h in (c) PBS, and (d) lipase solution. SEM images of (e) PCL sample after 24h growth with *E. coli*, (f) sonicated MoU-PCL sample after 2-week growth with *E. coli.* Scale bar: 500nm.

Section 3: Fabrication of PCL into different structures

Owing to the superior rheological and viscoelastic properties, PCL can be structured into different geometries, which enables its diverse applications, such as medical devices, tissue engineering[6]. **Figure S2** shows an example of shaping PCL into letters (CTH, abbreviation of Chalmers Tekniska Högskola) and round disks with different diameters.

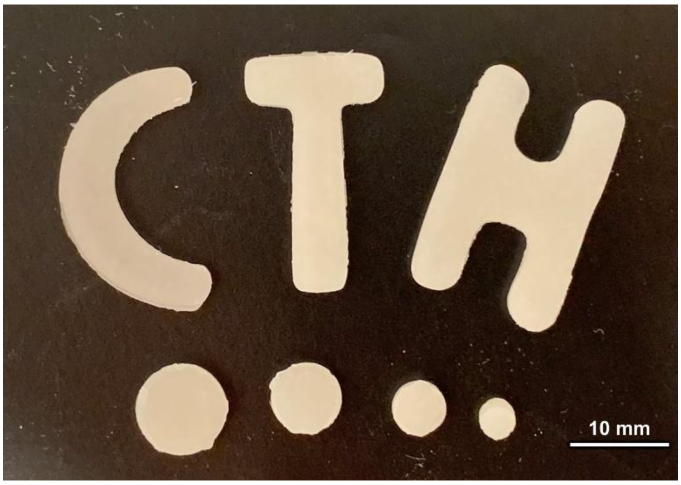

Figure S2 Images of PCL in different geometries

Section 4: MOF embedding with different solvents

Different solvents were applied for the MOF embedding, including acetone, DMF, and DCM. As shown in **Figure S3a**, after the treatment of DMF, PCL presented porous structures. After the treatment of DCM, PCL started to melt down, leading to curved surfaces around the edges and some cracks from the center (**Figure S3b**). These changes in the PCL surface led by DMF and DCM could bring uncontrollable variables in the assessment of antibacterial performance. Therefore, in this study, acetone was used for the MOF embedment, as smooth surfaces were observed after the treatment (main text, Figure 2b1).

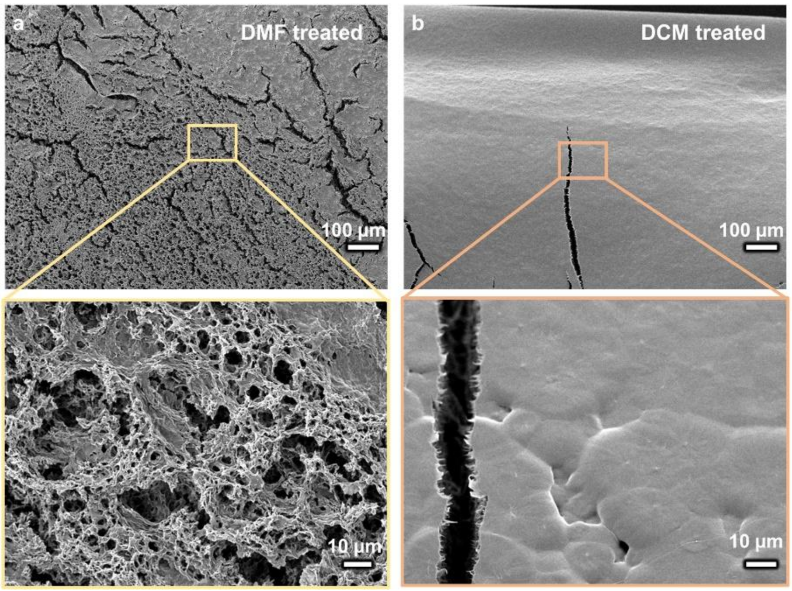

Figure S3 The SEM images of the PCL after the treatment of (a) DMF and (b) DCM

Section 5: Mechanical test of the PCL and MoU-PCL specimen

The tensile test was carried out for PCL and MoU-PCL specimen with 3 replicates, using a universal testing system (Instron). The fracture force was summarized in Table S1. The measured fracture force of the MoU-PCL composite (6.93 N) was approximately 93% of the pure PCL specimen (7.44 N). The mechanical performance of the PCL composite is highly relevant to their processing parameters and ratio of compositions.[7,8] Modifying the mechanical performance of the MoU-PCL would require a further investigation for certain applications.

Table S1 Fracture force of the PCL and MoU-PCL specimen

Fracture force (N)	PCL	MoU-PCL
**1**	7.70	6.52
**2**	7.34	6.96
**3**	7.27	7.31
**Average**	7.44	6.93
**Standard deviation**	0.19	0.32

Section 6: Microstructure of MOF-PCL composites via solvent casting

Three commonly reported solvents have been applied to achieve the MOF-PCL composites by solvent casting, including acetone, ethyl acetate, and DCM.[9,10] The microstructure of the MOF-PCL composites obtained from the solvent-casting approach was characterized by scanning electron microscopy (SEM). As shown in **Figure S4**, MOFs were immersed in the PCL matrix, and it was difficult to observe MOF nanostructures on the surface. Furthermore, some cracks and voids were observed on the MOF-PCL composites during the solvent evaporation process, as demonstrated in **Figure S4abc(2)**. These would introduce extra variables, such as surface area, for the analysis of antibacterial efficiency. Therefore, the MOF-PCL composites fabricated by the MOF embedment approach were used for the bactericidal experiments in this work.

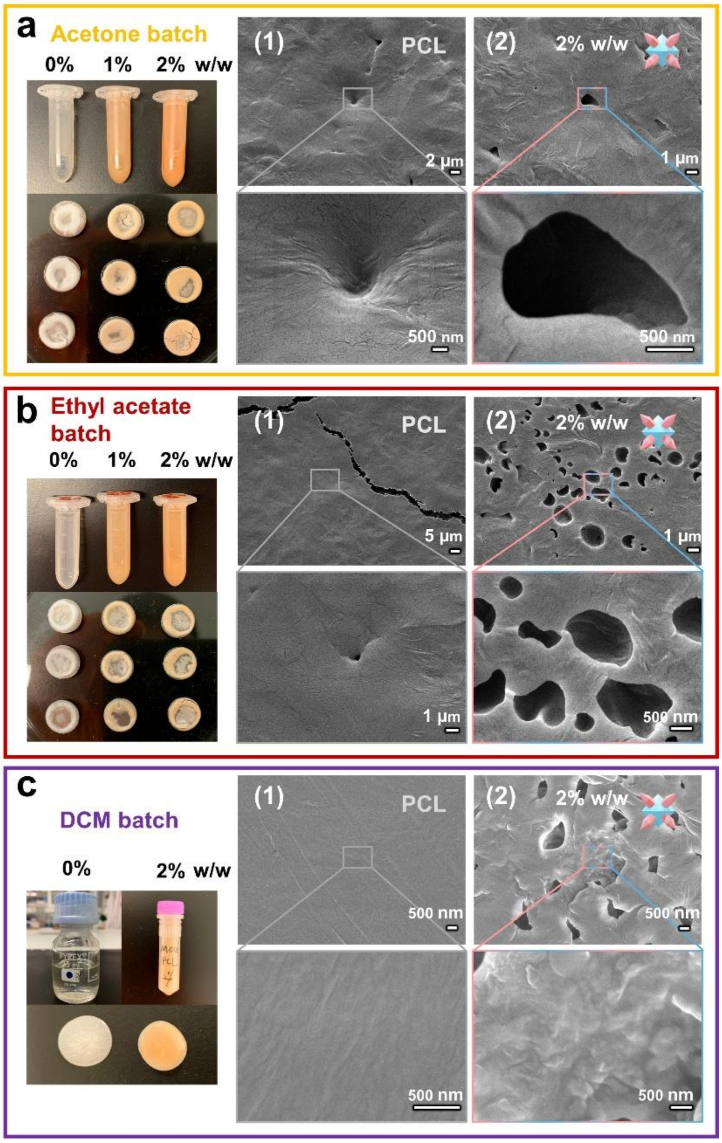

Figure S4 The photos and SEM images of the PCL and 2% w/w MOF-PCL composite obtained from the solvent casting method using different solvents: (a) acetone, (b) ethyl acetate, and (c) DCM. Cracks and voids were observed in the MOF-PCL composite by the solvent casting method.

Section 7: High-resolution XPS spectra for O *1s* and C *1s*

The high-resolution XPS spectra for O *1s* and C *1s* are shown in **Figure S5**. Two main peaks were observed in the O *1s* band (**Figure S5a**): O=C (O1) at 532.1 eV and O-C (O2) at 533.3 eV. Three main peaks were found in the C *1s* band (**Figure S5b**): C-C (C1) at 284.6 eV, C-O (C2) at 286.0 eV, and C=O(C3) at 288.6 eV. The O *1s* band and C *1s* band match the reported PCL XPS patterns.[11,12]

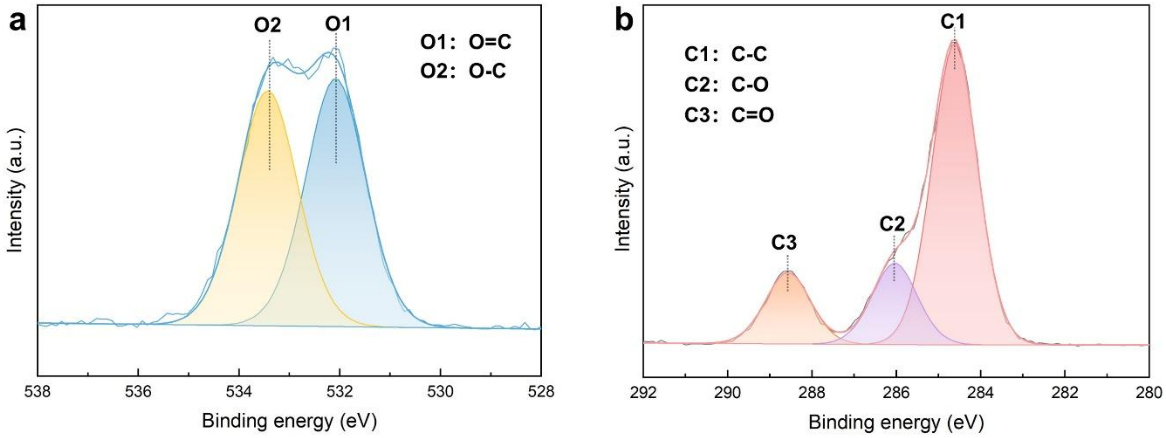

Figure S5 XPS spectra of (a) O *1s* and (b) C *1s*

Section 8: Water contact angle measurement

Wettability may influence bacterial adhesion.[13] In this work, the wettability of the obtained surfaces was characterized through water contact angle (CA) measurement. The CA values were recorded within 5 seconds once the water droplet landed on the surface. The hydrophobicity of the surface is MOFs as shown in **Table S2** and **Figure S6**. PCL (74°) and MoU-PCL composites (72°) presented a close range of water CA, which could be attributed to the shielding of the MoU particles by PCL after the MOF embedment.

Table S2 Water contact angle of obtained surfaces

Number	PCL (°)	MoU-PCL composite (°)
1	74	69
2	76	70
3	72	78
**Mean**	**74**	**72**
Standard deviation	2	5

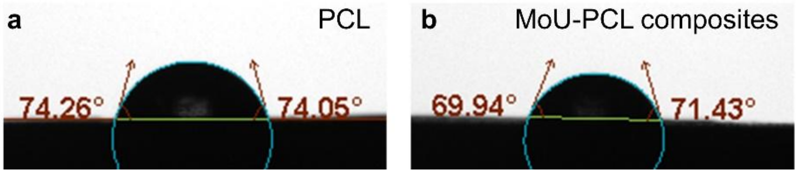

Figure S6 Images of water contact angle measurements for (a) PCL and (b) MoU-PCL composites

Section 9: Colony forming unit (CFU) results

The results of the CFU results are summarized in **Table S3**. These data were used for constructing the CFU bar chart in the main text (Figure 3).

Table S3 CFU results for *P. aeruginosa* and *S. epidermidis* for 24h and 72h growth

	***P. aeruginosa* 24 h (CFU/sample)**				***P. aeruginosa* 72 h (CFU/sample)**			
**Sample**	PCL	MoU-PCL	UiO66-PCL	MIL88-PCL	PCL	MoU-PCL	UiO66-PCL	MIL88-PCL
**CFU/ sample**	2.23E+09	2.72E+07	5.22E+08	3.05E+08	1.02E+09	2.33E+08	9.33E+08	4.67E+08
**Standard deviation**	3.20E+08	6.71E+06	9.83E+07	1.95E+08	3.30E+08	1.65E+08	3.70E+08	1.03E+08
	***S. epidermidis* 24 h (CFU/sample)**				***S. epidermidis* 72 h (CFU/sample)**			
**Sample**	PCL	MoU-PCL	UiO66-PCL	MIL88-PCL	PCL	MoU-PCL	UiO66-PCL	MIL88-PCL
**CFU/ sample**	2.65E+07	1.70E+06	4.62E+07	3.32E+06	5.98E+07	6.25E+06	6.33E+07	2.83E+07
**Standard deviation**	4.09E+06	4.82E+05	2.30E+07	1.38E+06	3.53E+06	3.60E+06	1.66E+07	5.31E+06

Section 10: MoU cytotoxicity on human cancer cells

To investigate the cytotoxicity of MoU exposure on human cells, the osteosarcoma cell line MG-63 was exposed to different concentrations of MoU for approximately 24 h before viability was measured. MG-63 cells (gifted by Lena Larsson at the Institute of Odontology, Sahlgrenska Academy, University of Gothenburg, Sweden) were resuspended in complete medium consisting of 90% Eagle's minimum essential medium (ATCC) with 10% heat-inactivated fetal bovine serum (Gibco) and seeded at a density of 10×10^3 cells in a total volume of 100 µl per well in a 96-wells plate with flat bottom. The plate was incubated at 37°C, 5% CO_2 and 95% relative humidity overnight. The following day the old medium was discarded and new medium with different concentrations (250 and 500 µg/ml) of MoU was added before plate was placed back in the incubator. Approximately 24 h after start of exposure the medium with MoU was removed from the cells, which was thereafter washed six times with Dulbecco's phosphate buffered saline (DPBS, Cytiva). Fresh medium was added to all wells, including wells not containing cells for a medium control, before 10 µl alamarBlue™ Cell Viability Reagent (Invitrogen) were added to all wells. Plate was incubated for 1 h followed by absorbance measurements at 570 and 600 nm in a spectrophotometer (Varioskan™ LUX Multimode Microplate Reader). Subsequent data analysis averaged the absorbance at 600 nm for the medium controls and subtracted them from the 570 nm readings for the test wells, as per manufacturer's instructions. Data are presented as percentage of the non-exposed cell control, with average value of four technical replicates in one biological replicate for each treatment.

Viability measurements show that MoU does not show any considerable cytotoxicity on human osteosarcoma MG-63 cells when used in solutions up to 500 µg/ml, as seen in **Figure S7**.

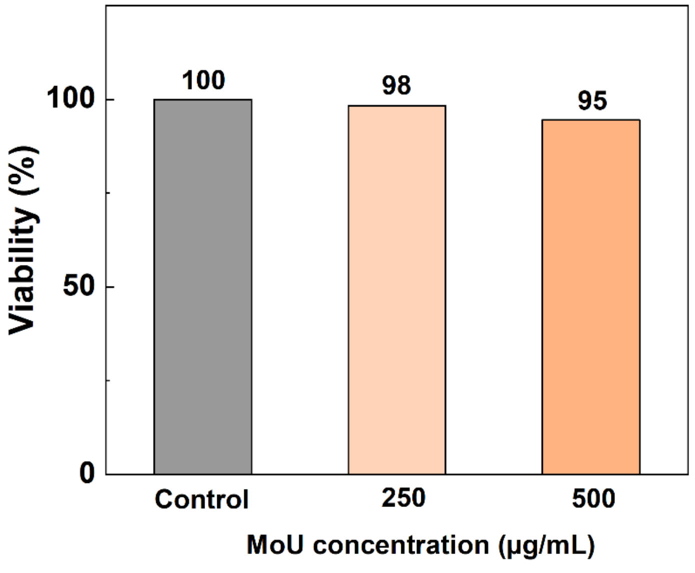

Figure S7 Viability of MG-63 cells after 24 h exposure of MoU, as measured with alamarBlue™ Cell Viability Reagent

Supporting references

(1) Cao, Z.; Pandit, S.; Noa, F. M. A.; Zhang, J.; Gao, W.; Rahimi, S.; Öhrström, L.; Mijakovic, I. Mechano-Bactericidal Surfaces Achieved by Epitaxial Growth of Metal-Organic Frameworks. arXiv March 20, 2025. https://doi.org/10.48550/arXiv.2503.16003.

(2) Cavka, J. H.; Jakobsen, S.; Olsbye, U.; Guillou, N.; Lamberti, C.; Bordiga, S.; Lillerud, K. P. A New Zirconium Inorganic Building Brick Forming Metal Organic Frameworks with Exceptional Stability. *J. Am. Chem. Soc.* **2008**, *130* (42), 13850–13851. https://doi.org/10.1021/ja8057953.

(3) Wang, X.-G.; Xu, L.; Li, M.-J.; Zhang, X.-Z. Construction of Flexible-on-Rigid Hybrid-Phase Metal–Organic Frameworks for Controllable Multi-Drug Delivery. *Angew. Chem. Int. Ed.* **2020**, *59* (41), 18078–18086. https://doi.org/10.1002/anie.202008858.

(4) Kwon, O.; Kim, J. Y.; Park, S.; Lee, J. H.; Ha, J.; Park, H.; Moon, H. R.; Kim, J. Computer-Aided Discovery of Connected Metal-Organic Frameworks. *Nat. Commun.* **2019**, *10* (1), 3620. https://doi.org/10.1038/s41467-019-11629-4.

(5) Pastorino, L.; Pioli, F.; Zilli, M.; Converti, A.; Nicolini, C. Lipase-Catalyzed Degradation of Poly(ε-Caprolactone). *Enzyme Microb. Technol.* **2004**, *35* (4), 321–326. https://doi.org/10.1016/j.enzmictec.2004.05.005.

(6) Woodruff, M. A.; Hutmacher, D. W. The Return of a Forgotten Polymer—Polycaprolactone in the 21st Century. *Prog. Polym. Sci.* **2010**, *35* (10), 1217–1256. https://doi.org/10.1016/j.progpolymsci.2010.04.002.

(7) Wan, C.; Chen, B. Poly(ε-Caprolactone)/Graphene Oxide Biocomposites: Mechanical Properties and Bioactivity. *Biomed. Mater.* **2011**, *6* (5), 055010. https://doi.org/10.1088/1748-6041/6/5/055010.

(8) Dwivedi, R.; Kumar, S.; Pandey, R.; Mahajan, A.; Nandana, D.; Katti, D. S.; Mehrotra, D. Polycaprolactone as Biomaterial for Bone Scaffolds: Review of Literature. *J. Oral Biol. Craniofacial Res.* **2020**, *10* (1), 381–388. https://doi.org/10.1016/j.jobcr.2019.10.003.

(9) Pappalardo, D.; Mathisen, T.; Finne-Wistrand, A. Biocompatibility of Resorbable Polymers: A Historical Perspective and Framework for the Future. *Biomacromolecules* **2019**, *20* (4), 1465–1477. https://doi.org/10.1021/acs.biomac.9b00159.

(10) Zheng, Q.; Li, J.; Yuan, W.; Liu, X.; Tan, L.; Zheng, Y.; Yeung, K. W. K.; Wu, S. Metal–Organic Frameworks Incorporated Polycaprolactone Film for Enhanced Corrosion Resistance and Biocompatibility of Mg Alloy. *ACS Sustain. Chem. Eng.* **2019**, *7* (21), 18114–18124. https://doi.org/10.1021/acssuschemeng.9b05196.

(11) Czwartos, J.; Nowak-Stępniowska, A.; Budner, B.; Fok, T.; Bartnik, A.; Wachulak, P.; Fiedorowicz, H. Polycaprolactone Scaffold Surface Modification with Soft X-Ray/Extreme Ultraviolet (SXR/EUV) Radiation and Low-Temperature Oxygen and Nitrogen Plasma for Biomedical Applications. *J. Mater. Sci.* **2024**, *59* (26), 11937–11951. https://doi.org/10.1007/s10853-024-09876-y.

(12) Ivanova, A. A.; Syromotina, D. S.; Shkarina, S. N.; Shkarin, R.; Cecilia, A.; Weinhardt, V.; Baumbach, T.; Saveleva, M. S.; Gorin, D. A.; Douglas, T. E. L.; Parakhonskiy, B. V.; Skirtach, A. G.; Cools, P.; Geyter, N. D.; Morent, R.; Oehr, C.; Surmeneva, M. A.; Surmenev, R. A. Effect of Low-Temperature Plasma Treatment of Electrospun Polycaprolactone Fibrous Scaffolds on Calcium Carbonate Mineralisation. *RSC Adv.* **2018**, *8* (68), 39106–39114. https://doi.org/10.1039/C8RA07386D.

(13) Linklater, D. P.; Baulin, V. A.; Juodkazis, S.; Crawford, R. J.; Stoodley, P.; Ivanova, E. P. Mechano-Bactericidal Actions of Nanostructured Surfaces. *Nat. Rev. Microbiol.* **2021**, *19* (1), 8–22. https://doi.org/10.1038/s41579-020-0414-z.
